# Oral Bisphosphonate Related Osteonecrosis of the Jaw: A Challenging Adverse Effect

**DOI:** 10.1155/2013/215034

**Published:** 2013-05-16

**Authors:** Ilke Coskun Benlidayi, Rengin Guzel

**Affiliations:** Department of Physical Medicine and Rehabilitation, Faculty of Medicine, Cukurova University, Adana 01330, Turkey

## Abstract

Oral bisphosphonates are the most commonly prescribed antiresorptive drugs for the treatment of osteoporosis. However, there are several adverse effects associated with oral bisphosphonates including the bisphosphonate related osteonecrosis of the jaw (BRONJ). With a better understanding of this side effect, reported incidences for BRONJ in oral bisphosphonate users have increased in time. The pathogenesis of BRONJ has not been well determined. Several risk factors such as dentoalveolar surgery, therapy duration, and concomitant steroid usage have been linked to BRONJ. Conservative and surgical methods can be preferred in the treatment. Preventative measures are of great importance for the patients at high risk. In this paper, osteonecrosis of the jaw secondary to oral bisphosphonates was reviewed in order to increase awareness as well as to renew the current knowledge.

## 1. Introduction

Oral bisphosphonates are synthetic drugs used primarily in the treatment of osteoporosis [1]. Since osteoporosis is the most common metabolic bone disease worldwide, there has been a remarkable increase in the prescription of the antiosteoporotic drugs including the bisphosphonates [2]. Being strong suppressors of osteoclasts, bisphosphonates slow the remodeling process and thus increase bone mineral density. Presently approved bisphosphonates for the treatment of osteoporosis have been shown to notably decrease the risk of fractures due to osteoporosis. Because of their confirmed effectiveness, bisphosphonates are considered as first line therapy in the treatment of osteoporosis and are the most widely prescribed antiresorptive agents [3].

Despite the benefits of these drugs, bisphosphonate related osteonecrosis of the jaw (BRONJ) which was first published in the literature in 2003 is a severe side effect of bisphosphonate therapy [4–7]. Since this initial identification, many cases have been reported [8–11].

## 2. Definition

In order to discriminate BRONJ from other conditions The American Society for Bone and Mineral Research define BRONJ as follows [12].

Patients may be considered to have BRONJ if they have all of the following criteria.

1. Current or previous treatment with a bisphosphonate;
2. exposed bone in the maxillofacial region that has persisted for more than eight weeks; and
3. no history of radiation therapy to the jaws.

However, stage 0 BRONJ where there is no exposed bone is not compatible with this definition. Therefore an update of the definition was performed as “exposed or otherwise necrotic bone” [13].

## 3. Pathogenesis

Despite the possibly linking theories, the pathogenesis of BRONJ has not been entirely understood. One of the underlying factors is the suppression of natural remodeling process due to the inhibition of osteoclasts. Remodeling is vital for bone healing and the suppression by bisphosphonates diminishes the healing capacity of the bone. It is not obvious whether bisphosphonates are found in the tissues at a high enough concentration to generate toxic effect on soft tissue in vivo, during normal oral administration. However, in vitro they produce direct toxic effect on the soft tissues of the oral cavity, which can also play a role in the development of BRONJ. Due to the antiangiogenic features of bisphosphonates, there may be a quantity of decline in the vascularity of bone. However, the histological studies have not endorsed this suggestion, thus reduction in vascularity seems not likely to have a significant role in the initiation of BRONJ. The particular occurrence of osteonecrosis due to bisphosphonates in the jaws area is in part related to the high turnover of alveolar bone as well as the exposure of jaw to the outside environment through the teeth and periodontal ligament [1, 14].

## 4. Epidemiology

Although there are several reports with regard to the incidence of BRONJ in the population receiving oral bisphosphonates the accurate incidence has not been well determined. BRONJ was reported to be 0.7/100,000 person/years of exposure [15]. Another study from Australia reported a frequency of 0.01–0.04% for patients treated weekly with alendronate [16]. The prevalence of BRONJ was reported as 0.06% in long-term oral bisphosphonate receivers [17]. 

BRONJ due to oral alendronate sodium was found to be more frequent than formerly reported with a prevalence of approximately 4% (9 out of 208 patients) [18]. An estimated prevalence of oral BRONJ was noted as 0.05–0.07% by the first report from Asia [19]. In a multicentre study from Europe 470 cases of BRONJ were evaluated and 37 were related with oral use (7.8%). The majority of those cases did not have any risk factors for BRONJ as defined by American Association of Oral and Maxillofacial Surgeons (AAOMS) [20]. A more recent report described an absolute risk ranging from 0.46% to 0.99% among patients receiving oral bisphosphonates [21]. Nevertheless, since some moderate and self-resolving cases are not recognized, establishing the incidence of oral BRONJ remains difficult.

## 5. Risk Factors

There are several risk factors for BRONJ that are categorized by AAOMS as follows.

### 5.1. Drug Related Risk Factors

*(a) Bisphosphonate Potency*. Since intravenous administration leads to a higher drug exposure than the oral route, osteonecrosis related to oral bisphosphonate therapy is less common than that of related to intravenous forms such as zolendronate which is also more potent than oral bisphosphonates [15]. Bisphosphonates are classified into two groups as nitrogen containing and non-nitrogen containing bisphosphonates. Non-nitrogen containing oral bisphosphonates include etidronate, tiludronate, and clodronate. Nitrogen containing oral bisphosphonates are alendronate, risedronate, and ibandronate. Nitrogen containing bisphosphonates possess higher risk regarding BRONJ development than non-nitrogen containing bisphosphonates [21]. It is not known if monthly dosing regimens have a distinctive role regarding the risk of BRONJ development when compared with weekly dosing [15]. In a multicentre study, the most frequent type of oral bisphosphonate was alendronate, followed by risedronate, ibandronate, and clondronate [20]. Diniz-Freitas et al. conducted a study in BRONJ patients receiving oral bisphosphonates [2]. Of the 20 patients 16 were taking alendronate, while 4 of them were on ibandronate. This higher incidence of alendronate associated osteonecrosis of the jaw may be related to the frequent presciription of alendronate, or can be explained by its dose, potency, half-time, and absorption related factors [22].

*(b) Duration of Therapy.* As oral bisphosphonates are less bio-available than intravenous formulations, they are used for long terms [20]. Longer duration (for more than two years) has been associated with an increased risk of oral BRONJ. According to a review identifying 103 patients of oral bisphosphonate (alendronate and risedronate) receivers, the mean time to the event until the onset of disease was 4.6 years. The median minimum time to the event was 3 years. This period is more than double of the 1.8 years that is found in zoledronic acid users. The fast development of BRONJ among intravenous bisphosphonate users is due to the higher and more rapid accumulation of these drugs in the bone [23]. Eighty-five osteoporotic patients using bisphosphonates who had been diagnosed with osteonecrosis of the jaw were identified in a systematic review. Of those patients 96.5% were taking oral bisphosphonates. The duration of the use of bisphosphonates was more than 1 year in 93.3% and more than 5 years in 40% [24]. In a 20 case series from Spain, mean interval between the initiation of therapy and confirmation of a BRONJ diagnosis was 66 months. However, in 7 patients the interval was less than 3 years [2]. Almăşan et al. reported that the risk of BRONJ was very high 12 months after oral administration in patients who had a trigger point in the jaws area [25]. Therefore, determination of BRONJ should be based not only the duration of the therapy but also other risk factors including local injury, systemic diseases, concomitant medication, and genetic predisposition [2].

### 5.2. Local Risk Factors

 Dentoalveolar surgical procedures such as teeth extractions, periapical surgery, dental implant placement, and periodontal surgery involving osseous injury, as well as concomitant oral disease and poor oral hygiene, are risk factors for BRONJ. Additionally, as bisphosphonates have been shown to inhibit the proliferation of keratinocytes in the oral mucosa, injury of the oral mucosa due to the poorly fitting removable dental prostheses may increase the risk of BRONJ in bisphosphonate users. BRONJ due to oral bisphosphonates may occur spontaneously or more commonly after a trauma to the bone. When there is increased necessity for bone repair following a trauma, the inhibition of remodeling due to bisphosphonate therapy makes the bone vulnerable to necrosis [2, 26]. According to the results of a review including 11 publications reporting 26 cases of BRONJ in patients receiving bisphosphonates for osteoporosis, 15 patients had history of invasive dental procedure, 12 (80%) had undergone dental surgery or experienced dental trauma at the site of BRONJ. Another retrospective review revealed that the risk of BRONJ is very high twelve months after oral uptake in patients who have a trigger point in the jaws area such as dental extractions, irrecoverable teeth-foci, prosthetic injuries, and surgical procedures [25]. Villa et al. reported that, patients with osteoporosis receiving bisphosphonates may develop osteonecrosis of the mandible, especially in the presence of an active infectious process in the mouth such as periodontal disease or suppuration. In addition, patients who were on bisphosphonate treatment and had dental extraction were found to be 3 times more likely to develop BRONJ [27]. Local anatomy also plays a part in the development of BRONJ. Lesions are more frequent in the mandible than maxilla and more common in bony prominences with thin mucosa. In a trial consisting 30 oral BRONJ patients, 27 of them occurred in the mandible while only one case occurred in the maxilla [22].

### 5.3. Demographic and Systemic Factors

Increased age (older than 65 years) was found to be a risk factor for BRONJ. Additionally, oral BRONJ is more common in women than men. For instance, all of the participants of a study about oral BRONJ were women [22]. This is not an unexpected finding, since osteoporosis—particularly the postmenopausal type—is the primary indication for oral bisphosphonates. Some systemic factors such as renal dialysis, low hemoglobin, obesity, immunosuppression, rheumatoid arthritis, smoking, and diabetes were reported to increase the risk for BRONJ [15, 28, 29]. Additionally, prednisone and methotrexate used in autoimmune diseases are associated with an additional inhibition of remodeling in oral bisphosphonate cases [22]. 

### 5.4. Genetic Factors

 Single nucleotide polymorphisms in the cytochrome P450-2C gene and IGF 1 genes may play part in the pathogenesis of BRONJ [30, 31].

### 5.5. Preventative Factors

 According to the recommendation of the AAOMS Taskforce on BRONJ, undergoing dental evaluations and getting required treatment before being started on bisphosphonates may be effectual on decreasing the risk for BRONJ [15].

## 6. Clinical Presentation and Other Diagnostic Findings

Patients with oral BRONJ may be symptomatic or asymptomatic. Without any declared symptoms, they may present with exposed alveolar bone during routine dental evaluations. Patients may also present with symptoms such as pain and evidence of local or widespread infections. The patients may state a trauma due to dental prosthesis or a former dental procedure whereas some cases do not have an obvious preceding factor [14]. Panoramic radiographs, dental cone beam or spiral computed tomographies can be used to detect BRONJ. However, panoramic and periapical radiographs may not be as helpful in identifying the changes in the initial phase of osteonecrosis. Similarly, computerized tomography has not been proved to be useful in early detection of asymptomatic BRONJ patients. When there is no clinically exposed bone, scintigraphy, PET scan, or MRI may identify early areas of bony involvement [1, 3]. Serum C-telopeptide crosslink of type 1 collagen (CTX) is a marker used for predicting the risk of BRONJ. However its role as a predictive marker of BRONJ remains controversial [20].

## 7. Stages

According to the BRONJ staging which was revised in the AAOMS position paper in 2009, asymptomatic patients who have been treated with oral or intravenous bisphosphonates and have no apparent necrotic bone are at risk category. However, non-specific symptoms or clinical and radiographic findings and without a clinical evidence of necrotic bone refer to stage 0. Asymptomatic patients having an exposed and necrotic bone, but no evidence of infection are regarded as stage 1. Stage 2 is corresponded to a situation including exposed and necrotic bone associated with infection. Exposed and necrotic bone in patients with pain, infection, and one or more of the following: exposed and necrotic bone extending beyond the region of alveolar bone, resulting in pathologic fracture, extra-oral fistula, oral antral/oral nasal communication, or osteolysis extending to the inferior border of the mandible of sinus floor refers to stage 3 [15].

## 8. Management

The primary aim of BRONJ management is prevention of infection of the necrotic bone and reduction of symptoms. Patients with oral BRONJ generally have mild clinical appearances of necrosis and respond better to treatment strategies. Regarding the patients at risk category no treatment is indicated. However, patient education in terms of clinical manifestations and symptoms of potential BRONJ is crucial. These patients should be advised to take an opinion of an expert in case of any concern of developing BRONJ. Stage 0 is managed with drugs for pain when indicated. In the first stage, where no sign of inflammation is present, antimicrobial mouth rinses are recommended. Patients at stage 1 should be evaluated on a quarterly basis. Since abandoning the oral bisphosphonate therapy provides gradual improvement in clinical disease, indications for bisphosphonate treatment must be reviewed. Antibacterial mouth rinses are prescribed in order to avoid infection of exposed bone. If there is any evidence of infection (i.e., stage 2 and 3), in addition to mouth rinses, treatment with oral antibiotics is essential. Some refractory cases may require combination antibiotic therapy. In order to remove necrotic bone and create soft tissue coverage of remaining healthy bone, surgical procedures are performed in stages 2 and 3. Superficial debridement in order to alleviate soft tissue irritation is recommended for stage 2. Spontaneous sequestration or resolution following debridement surgery can be achieved by discontinuation of oral bisphosphonates. However, if systemic conditions permit, modification or cessation of oral bisphosphonate therapy should be considered. Since bisphosphonates affect the whole jaw and surgical trauma to the bone could lead to progression of osteonecrosis, aggressive surgical treatment approach remains controversial. However, radical surgical management including resection is advocated for longer term palliation of infection and pain where there are large segments of necrotic bone or pathological fractures of the bone (stage 3). 

Suggested additional therapies for BRONJ include hyperbaric oxygen, parathyroid hormone, platelet rich plasma and lasers [1, 14, 15]. Since hyperbaric oxygen is known to decrease edema and inflammation, enhance microbial killing, vasculogenesis and tissue repair in wounds, it can improve wound healing and pain scores when added to surgical and non-surgical treatment protocols [32]. The efficacy of parathyroid hormone in BRONJ mostly depends upon its ability to elevate suppressed bone remodeling associated with bisphosphonates. However, its use in patients with metastatic cancer should be considered meticulously as it may promote skeletal metastases [33, 34]. The efficacy of these additional therapies should be established entirely by further researches. 

## 9. Prevention

Prevention strategies aim to lower the risk of BRONJ. Since the risk of oral BRONJ appears to rise in treatment periods longer than 3 years, reduction this period may be helpful in patients with comorbidities such as chronic corticosteroid use. A patient with poor dental health before starting on oral bisphosphonates should be examined by a dentist in order to optimize the oral hygiene. In case of any oral surgical treatment, strategy should be based on the duration of bisphosphonate therapy, as well as concomitant drug use. No adjustment or interruption in the premeditated oral surgery is necessary for patients who have been on oral bisphosphonates for less than 3 years and have no clinical risk factors. There is also no need to evaluate the bone turnover marker levels such as serum CTX. However, patients who underwent dental implant placement procedures should be followed up regularly. Utilizing CTX along with a drug holiday or altering the dosing can also be considered. If concomitant use of steroids is present in a patient receiving oral bisphosphonates for less than 3 years whose systemic condition persist, drug holiday for at least 3 months prior to oral surgery is recommended [15]. Although CTX is not an ultimate predictor of BRONJ, it might be useful regarding the estimation of risk prior to a surgical procedure [35]. Deferring the planned surgery and having a drug holiday are recommended by Marx et al. if the CTX value is less than 150 pg/mL. However, when CTX level is 150 pg/mL or higher, it is suggested that the patients can undergo invasive oral surgery with a minimum risk of osteonecrosis [22]. Following the surgery, bisphosphonates should not be prescribed till bony healing. Similarly, in cases who have been receiving oral bisphosphonate therapy for more than 3 years with or without any simultaneous prednisone or other steroid drugs, administration of oral bisphosphonates should be stopped 3 months prior to oral surgery, if systemic conditions permit [15]. However Marx et al. do not limit oral surgery, if CTX is 150 pg/mL or above. The bisphosphonate can be prescribed again when the osseous healing occurs [15, 22]. On the other hand, due to lack of evidence, stopping bisphosphonates prior to invasive dental procedures remains controversial [36]. In addition, the precise duration of pathological effects of bisphosphonates awaits to be elucidated as bone turnover markers remain suppressed for several years following discontinuation of bisphosphonate treatment [20]. According to the American Dental Association, the decision of bisphosphonates' discontinuation should be based primarily upon the risk for skeletally related events such as fractures and secondary to low bone density, not the feasible risk of BRONJ. Additionally the use of serum CTX as a resorption marker is not endorsed by the American Dental Association [37]. 

## 10. Prognosis

Although there is not an accurate judgment regarding the prognosis of BRONJ, the clinical course and outcome of BRONJ is suggested to be associated with the antecedent factors and comorbid status. In a recent study of 30 cases of oral BRONJ, while patients with spontaneous BRONJ presented with focal disease generally healed within 8 months, patients developing BRONJ after tooth extraction healed in 18 months and those with relevant comorbidities had a lower probability of healing and a longer median time to healing (20 months) [38].

## 11. Conclusion

Bisphosphonates are one of the most prescribed drugs all around the world. Although it is a rare condition, potential risk of BRONJ due to oral bisphosphonates should not be neglected. Of the patients on oral bisphosphonates, those with appropriate dental care do not require a dental examination prior to the initiation of treatment. However, patients who are not receiving regular dental care or with concomitant risk factors such as cancer, corticosteroids, chemotherapy, and poor oral hygiene should undergo a broad oral examination by a dentist either prior to or following the initiation of bisphosphonates [26]. An oral health program in terms of oral hygiene methods and routine dental care is considered as the most favorable approach [39]. Stopping smoking and limiting the alcohol intake are also recommended. Patient education in terms of the symptoms and initial signs of BRONJ is essential. If diagnosed timely, outcomes of BRONJ treatments are favorable. Therefore, detailed regular intraoral examinations are crucial for detecting the early stages of BRONJ lesions [40]. In cases on oral bisphosphonates and with concomitant risk factors, avoiding invasive dental surgery when possible is also recommended. 

Eventually, it is of great importance for physicians dealing with osteoporosis such as physiatrists, endocrinologists, and rheumatologists to be aware of the potential risk of developing BRONJ in patients on oral bisphosphonates and to work in accordance with dentists. 
